# GnRh agonists as precipitating components of psychiatric pathology. A case report.

**DOI:** 10.1192/j.eurpsy.2023.2211

**Published:** 2023-07-19

**Authors:** A. Guerrero Medina, J. S. García Eslava, A. C. Martín Rodriguez, L. Martinez Salvador, M. J. Alvarez Alonso, M. Aubareda Magriña

**Affiliations:** Psychiatry, Consorci Hospitalari de Vic, Vic, Spain

## Abstract

**Introduction:**

GnRh agonists are drugs used in various gynecological pathologies, among which is endometriosis. They act by stimulating GnRh receptors in the pituitary gland. This sustained and continuous stimulation of GnRh, will initially generate an increase in the release of luteinizing hormones and follicle-stimulating hormones, subsequently losing sensitivity to the receptors, internalizing them, and thus suppressing the release of these hormones, which would entail an ovarian suppression, thereby inhibiting the release of estrogens and progesterone. Psychiatric adverse effects have been described. Gonzalez-Rodriguez et al (Front Psychiatry 2020; 11:479), described this association with changes in mood, and the presence of a series of cases where the link between GnRh agonist and the possibility of presenting psychotic symptoms is observed. Wieck (Curr Top Behav Neurosci 2011;8:173-87), Frokjaer (J Neurosci Res 2020;98(7):1283-1292), Brzezinski-Sinai et al (Front Psychiatry 2020;11:693) reported that this association could be related with the relationship of the hypothalamic-pituitary-gonadal axis, hormonal fluctuation and its relationship with the dopaminergic regulation, a genetic component that would increase the predisposition to trigger psychiatric pathology in patients with greater sensitivity to hormonal fluctuations, and the loss of neuroprotection generated by the decrease of estrogens in the central nervous system. All of this in the context of multiple environmental and genetic factors that participate together in the appearance of the disease.

**Objectives:**

To describe the importance of detecting the risk factors that can precipitate a psychotic episode, including the use of certain drugs, such as GnRh agonists.

**Methods:**

We describe a case of a 45 year old patient with endometriosis with multiple organ involvement who went to the emergency room due to behavioral changes in the context of a brief psychotic disorder with “ad-integrum” recovery.

**Results:**

A retrospective analysis of the case is conducted, observing an association between the introduction of GnRh agonists and the presentation of a first psychotic episode.

**Conclusions:**

The importance of this case lies in the limited evidence of this association in the literature, and the implication of these drugs in the triggering of psychiatric pathology, being an aspect to be considered by psychiatrists in their patient’s follow-up.

**Disclosure of Interest:**

None Declared

